# Analytical Sensitivity Analysis and Clinical Impact Modeling of Rapigen Rapid Diagnostic Tests for Malaria

**DOI:** 10.4269/ajtmh.24-0003

**Published:** 2024-09-03

**Authors:** Allison Golden, Hannah C. Slater, Ihn Kyung Jang, Sayali Walke, Thanh T. Phan, Greg T. Bizilj, Andrew Rashid, Rebecca Barney, Smita Das, Melissa J. Rist, James S. McCarthy, Francois Nosten, Jordi Landier, Mallika Imwong, Jennifer C. C. Hume, Issaka Sagara, Sara A. Healy, Patrick E. Duffy, Henry Ntuku, Davis Mumbengegwi, Michelle S. Hsiang, Sean C. Murphy, John Rek, Katherine Torres, Dionicia Gamboa, Gonzalo J. Domingo

**Affiliations:** ^1^ PATH, Seattle, Washington;; ^2^ Queensland Institute of Medical Research, Berghofer Medical Research Institute, Brisbane, Australia;; ^3^ Department of Infectious Diseases, The Peter Doherty Institute for Infection and Immunity, The University of Melbourne, Melbourne, Australia;; ^4^ Shoklo Malaria Research Unit, Mahidol–Oxford Tropical Medicine Research Unit, Faculty of Tropical Medicine, Mahidol University, Mae Sot, Thailand;; ^5^ Centre for Tropical Medicine and Global Health, Nuffield Department of Medicine, University of Oxford, Oxford, United Kingdom;; ^6^ Aix Marseille University, l’Institut de recherche pour le développement (IRD), L’Institut national de la santé et de la recherche médicale (INSERM), Sciences économiques et sociales de la santé & traitement de l’information médicale (SESSTIM), Institut des sciences de la santé publique (ISSPAM), Aix Marseille Institute of Public Health, Marseille, France;; ^7^ Department of Molecular Tropical Medicine and Genetics, Faculty of Tropical Medicine, Mahidol University, Bangkok, Thailand;; ^8^ Laboratory of Malaria Immunology and Vaccinology, National Institute of Allergy and Infectious Diseases, National Institutes of Health, Bethesda, Maryland;; ^9^ Malaria Research and Training Center, University of Sciences, Techniques and Technologies of Bamako, Bamako, Mali;; ^10^ Malaria Elimination Initiative, Institute for Global Health Sciences, University of California, San Francisco, California;; ^11^ Malaria Operational Research Program, Centre for Research Services at the University of Namibia, Windhoek, Namibia;; ^12^ Department of Laboratory Medicine and Pathology, University of Washington, Seattle, Washington;; ^13^ Infectious Diseases Research Collaboration, Kampala, Uganda;; ^14^ Departamento de Ciencias Celulares y Moleculares, Facultad de Ciencias e Ingeniería, Universidad Peruana Cayetano Heredia, Lima, Peru

## Abstract

Laboratory benchmarking allows objective analysis of the analytical performance of malaria rapid diagnostic tests (RDTs). We present the analytical detection limits of the Rapigen BIOCREDIT Malaria Ag Pf/Pv (pLDH/pLDH), the Rapigen BIOCREDIT Malaria Ag Pf (pLDH/HRPII), and two best-in-class WHO-prequalified comparator RDTs, generated using standardized panels containing recombinant antigen, in vitro cultured parasites, international standards, and clinical samples. Detection limit antigen concentrations of HRP2, PfLDH, and PvLDH were determined for the Rapigen and comparator RDTs. Detection of antigens in international units (IU)/mL was also evaluated. The Rapigen Ag Pf (pLDH/HRPII) detected 3.9 and 3.9 IU/mL for PfLDH and HRP2, respectively, and the Ag Pf/Pv (pLDH/pLDH) detected 3.9 and 5.0 IU/mL for PfLDH and PvLDH, respectively. The comparator HRP2/PfLDH and HRP2/PvLDH detected 15.6 and 31.3 IU/mL for HRP2 and PfLDH and 15.6 and 50.0 IU/mL for HRP2 and PvLDH, respectively. The RDT clinical sensitivity was predicted through application of analytical detection limits to antigen concentration distributions from clinical symptomatic and asymptomatic cases. Febrile cases would be detected in a majority by both standard and Rapigen RDTs, but incremental increases in sensitivity in the Rapigen RDTs may be important for clinical cases currently missed by microscopy. Rapigen RDTs were predicted to have improved detection of asymptomatic cases and infections with parasites carrying hrp2 deletions through more sensitive PfLDH detection. Through the benchmarking and simulation of clinical sensitivity, a method for rapidly assessing the ability of new RDTs to meet clinical needs using high-sensitivity antigen distribution data is presented.

## INTRODUCTION

Rapid diagnostic tests (RDTs), typically in the form of lateral flow cassettes, are the primary means of diagnosing malaria, alongside microscopy. In the absence of an expert microscopist, RDTs fulfill the need of clinics to have a fast and cost-effective method of diagnosis.

Malaria RDTs function by detecting protein antigens, produced by the malaria parasite, which are circulating in the peripheral blood. Rapid diagnostic tests used to diagnose *Plasmodium falciparum* (Pf) malaria in the vast majority detect the Pf-specific histidine-rich protein 2 (HRP2), whereas RDTs used to diagnose either *Plasmodium vivax* (Pv) or malaria caused by any human *Plasmodium* species target the conserved essential enzyme lactate dehydrogenase (LDH). Although HRP2 has been the most sensitive marker to date for Pf infection, there are two main challenges to its ongoing use as the sole biomarker in diagnosis of Pf infection. First, HRP2 can remain in the circulation after treatment and persist for weeks beyond clearance of the parasite, resulting in possible false-positive results in individuals who have recently been treated for malaria.^1^^–^^3^ Second, the Pf parasite remains viable with gene deletions in *hrp2* and the highly homologous *hrp3.* Pf with *hrp2*/*hrp3* deletions has led to the clinical presentation of individuals with false-negative RDT results, even in symptomatic cases presenting with high parasite densities.^1^ Such deletions have prompted dedicated WHO guidance as well as surveillance strategies to monitor their prevalence.^2^^,^^3^ Although first detected in Peru, *hrp2*/*hrp3* deletion surveillance and clinical data point to increases of *hrp2*/*hrp3* deletion prevalence, higher than 20%, in many countries^4^ from diverse malaria geographies of the world, including in Peru, countries in the Horn of Africa, and India. If significant prevalences of *hrp2*/*hrp3* gene deletions are observed, HRP2-based diagnostics become inappropriate for use.^5^ Instead, an RDT that also detects *P. falciparum*–specific LDH (PfLDH) may be required, but observed trade-offs in sensitivity compared with detection of Pf through HRP2 further highlight why advances in LDH sensitivity are needed.^6^ HRP2 has been a more robust antigen than PfLDH due to better assay binding kinetics of antibodies against HRP2 compared with PfLDH (and PvLDH) and higher expression of HRP2 relative to pfLDH.

Detection of Pv infection currently relies solely on *P. vivax*–specific LDH (PvLDH). Detection of Pv is further challenged by lower parasite densities and circulating parasites than Pf—and correspondingly lower blood antigen levels—common with Pv infections.^7^ As a result, RDTs for diagnosing Pv have poorer sensitivity than tests for Pf employing HRP2 detection. Gains have been made in the limits of detection of HRP2 and, more recently, pLDH.^8^^,^^9^ Understanding what such gains mean for clinical performance requires analysis of incremental gains in sensitivity, comparison to clinical data, and understanding of the distributions of antigen levels.

A benchmarking protocol was developed to characterize the analytical sensitivity of malaria RDTs for Pf and Pv. The benchmarking panel consists of well-characterized protein antigen sources such as recombinant protein, diluted clinical samples, and cultured parasites in which LDH and HRP2 have been quantified. This benchmarking protocol was applied to two WHO-prequalified RDTs as well as two RDTs produced by Rapigen: one RDT for Pf, BIOCREDIT Malaria Ag Pf (pLDH/HRPII) with test lines to detect HRP2 and PfLDH; and one for Pf and Pv, the BIOCREDIT Malaria Ag Pf/Pv (pLDH/pLDH) with test lines to detect PfLDH and PvLDH. The analytical performance is described, and their clinical performance is predicted through modeling.

## MATERIALS AND METHODS

### Benchmarking panels materials.

Whole blood units taken in dipotassium EDTA from five healthy donors were used for dilution of panel members (Interstate Blood Bank, Memphis, TN). The individual negative samples were evaluated using the Q-Plex™ Human Malaria (5-Plex) (Quansys Biosciences, Logan, UT) to confirm that they did not have detectable malaria antigen. The five units were then combined in equal volumes to prepare a pooled negative blood sample. Recombinant HRP2 tagged with glutathione-S-transferase protein was purchased from Microcoat Biotechnologie (Starnberger See, Germany). Recombinant PfLDH and PvLDH proteins were purchased from MyBioSource (San Diego, CA). Human recombinant LDH was acquired from the University of Queensland Protein Expression Facility (St. Lucia, Australia). The WHO international standards for Pf (product code 16/376) and Pv (product code 19/116) antigens were purchased from the National Institute for Biological Standards and Control (NIBSC; Hertfordshire, United Kingdom). Pf W2 (*hrp2+*/*hrp3+*), D10 (*hrp2–*/*hrp3+*), HB3 (*hrp2+*/*hrp3–*), and Dd2 (*hrp2–*/*hrp3+*) strains were obtained from the Biodefense and Emerging Infections Research Resources Repository (Manassas, VA) and the 3BD5 (*hrp2–*/*hrp3–*) strain was obtained from the National Institute of Allergy and Infectious Diseases (Bethesda, MD). *Plasmodium knowlesi* strain A1-H2 was a gift from Dr. Rob Moon (London School of Hygiene and Tropical Medicine, United Kingdom). Clinical *P. falciparum*– and *P. vivax*–positive samples used to prepare panels were obtained from Discovery Life Sciences (Santa Barbara, CA).

### Recombinant protein panel members and international standards.

Recombinant HRP2-GST and pLDH stock solution concentrations were confirmed by amino acid sequencing (University of Nebraska Medical Center Protein Structure Core Facility, Omaha, NE). Proteins were diluted serially into pooled negative whole blood and aliquoted for storage at −80°C. Then, NIBSC standards 16/376 and 19/116 were reconstituted according to instructions and then serially diluted into the pooled negative whole blood and aliquoted for storage at −80°C.

### Culture panel members.

*Plasmodium falciparum* strains ITG, Dd2, D10, HB3, and 3BD5 and *P. knowlesi* were prepared at PATH using in vitro culture and synchronization methods previously described.^10^^–^^14^ Parasite count was determined via staining of smear and 100× oil immersion objective light microscopy. Parasitized red blood cells were washed with phosphate-buffered saline and pelleted and frozen for long-term storage at −80°C until serially diluted for use in panels. The Pf culture panel containing cultured W2 was purchased from ZeptoMetrix (product code KZMC043, ZeptoMetrix, Franklin, MA).

### Clinical dilution panel members.

All clinically positive samples from Discovery Life Sciences used in panels were confirmed positive for *Plasmodium* monoinfection by photo-induced electron transfer polymerase chain reaction (PET-PCR).^15^ Briefly, DNA was extracted from 100 µL of whole blood samples using the QIAamp DNA Mini Kit (Qiagen Inc., Chatsworth, CA). *Plasmodium* genus–specific PET-PCR was performed in duplicate using 5 µL of DNA. Positive controls for PET-PCR consisted of samples with cultured 3D7 Pf or the plasmid for Pv (R64, kindly donated by the U.S. CDC), and negative control of nuclease-free water were included in each run. Before pooling, each clinical sample was characterized for antigen concentration by QPlex. Dilutions of clinical Pf-positive pools were prepared by combining five individual Pf-positives, then serially diluting into pooled negative whole blood. Dilutions of clinical Pv-positive pools were prepared by combining five individual Pv-positives, then serially diluting into pooled negative whole blood. Additionally, 10 individual Pf-positive and 10 Pv-positive samples were each serially diluted to prepare panels.

### Antigen quantification.

All panel members were analyzed for malaria antigen concentration to quantify simultaneously malaria proteins HRP2, PvLDH, pan-malarial LDH, PfLDH, and human C-reactive protein.^16^^,^^17^ Each sample was tested both neat and diluted 50-fold to improve the dynamic range of the quantification. Preparation of calibrators and samples and all other steps were conducted according to the protocol. Image collection and analysis were performed using the Q-View Imager Pro and Q-View™ analysis software (Quansys Biosciences).

### Malaria benchmarking panels.

A list of the panel members is shown in Table 1. For each panel member, a categorical classification relevant to test performance is listed, and the number of serial dilution steps is shown. Most panels target concentrations that span the limits of detection of both conventional and more highly sensitive RDTs. The target analytes relevant to each panel member are also listed.

**Table 1:** Composition of malaria benchmarking panel

Panel Member Series	No. of Panel Members in Series	Target Analytes
**Recombinant Protein**		
Recombinant HRP2, GST tag	10	HRP2
Recombinant PfLDH, His tag	8	PfLDH
Recombinant PvLDH, His tag	8	PvLDH
Recombinant human LDH, His tag	3	Specificity for pLDH
**International standards**		
NIBSC Pf antigen standard, 16/376	8	HRP2, PfLDH
NIBSC Pv antigen standard 19/116	12	PvLDH
**Cultured parasites**		
*P. falciparum*, ITG *P. falciparum*	10	HRP2, PfLDH
3BD5 (*hrp2−*/*hrp3−) P. falciparum*	6	PfLDH
*P. falciparum* culture panel, W2	7	HRP2, PfLDH
D10 (*hrp2−*/*hrp3+) P. falciparum*	5	HRP2, PfLDH
Dd2 (*hrp2−*/*hrp3+) P. falciparum*	5	HRP2, PfLDH
HB3 (*hrp2+*/*hrp3−) P. falciparum*	5	HRP2, PfLDH
Cultured *P. knowlesi*, PATH	8	Specificity for Pf or PvLDH Reactivity to *P. knowlesi* LDH
**Clinical specimens**		
Clinical Pf+ pool dilutions, Pf parasite clinical pool of five positive individuals	8	HRP2, PfLDH
Clinical Pv+ pool dilutions, Pv parasite clinical pool of five positive individuals	8	PvLDH
Negative bloods, pooled and individual	5	Specificity overall
10 individual Pf clinical specimens, diluted 20-fold to 6,000-fold	10 × 10	HRP2, PfLDH
10 individual Pv clinical specimens, diluted 10-fold to 800-fold	10 × 7	PvLDH

GST = glutathione-S-transferase; HRP2 = histadine-rich protein 2; LDH = lactate dehydrogenase; NIBSC = National Institute for Biological Standards and Control; Pf = *Plasmodium falciparum*; pLDH = *Plasmodium* lactate dehydrogenase; Pv = *Plasmodium vivax*.

Panel member series descriptions are listed under categories of recombinant protein, international standards, cultured parasites, or clinical specimens, along with the number of dilutions and/or individuals per series.

### Tests used in evaluation.

Tests used in the evaluation were from Rapigen Inc. (Suwon, South Korea): Rapigen BIOCREDIT Malaria Ag Pf/Pv (pLDH/pLDH) containing test lines to detect PfLDH and PvLDH, and the Rapigen BIOCREDIT Malaria Ag Pf (pLDH/HRPII) containing test lines to detect Pf HRP2 and PfLDH. Comparators for Pf and Pv infection were two best-in-class WHO-prequalified tests containing HRP2 and PfLDH detection lines and HRP2 and PvLDH detection lines.

### Evaluation of RDTs using malaria benchmarking panels.

Benchmarking panel member aliquots were kept frozen at −80°C and thawed for up to 2 hours before use and not refrozen or reused after testing. Blood samples were added to tests by calibrated pipettor, in the volume recommended in instructions for use (5 µL). Each test type was run with panel members using three to five replicates for all panel proteins used in testing specificity, and for decreasing concentrations of positive panel members that had adjacent positive results until a clear pattern of negativity was reached with lower concentrations of two or more adjacent dilutions testing negative for 100% of replicates. Concentrations near detection limit were chosen for specific panel members and run with a total of 40 replicate tests above, at, and below concentration identified as near limit of detection. Exceptions to this increased resolution in testing were for positives generated with human recombinant LDH, clinical pool dilutions, clinical individual samples, or culture-derived panel members with limited quantities. Test line intensity was assigned based on comparison to an intensity scale card provided by the manufacturer. Any visible test line was considered positive. All test results were interpreted at times according to manufacturer instructions. If a range of time was given, the earliest time point in the range was used. Test line signal intensity was measured by comparison to a manufacturer-provided scale. All invalid tests were recorded. For each test run, qualitative observations of problems with flow quality, line quality, high background, or test readability were noted.

### Clinical samples used in performance modeling.

Human specimens, from which reference data were used in this study, were received from a commercial supplier Discovery Life Sciences (Huntsville, AL), from the Foundation for Innovative New Diagnostics (Geneva, Switzerland) Specimen Bank, or collected in malaria research studies. All clinical samples used in the study were frozen whole blood samples. Approvals for these studies for collection of specimens and ongoing malaria research were granted by the following institutional review boards (IRBs), with identification number listed in parentheses: QIMR Berghofer Medical Research Institute, Brisbane, Australia, Human Research Ethics Committee (2080, 2092, 2098, and 2142, and ACTRN12617000048381); Oxford Tropical Research Ethics Committee, OxTREC, Tak Community Advisory Board, Myanmar, and by the relevant village committee (516-17, 1017-13 and 1015-13); Mali Faculté de Médecine de Pharmacie et d’Odonto Stomatologie, Bamako, Mali and National Institute of Allergy and Infectious Diseases, National Institutes of Health, Bethesda, MD, USA (NCT02334462); Namibia Ministry of Health and Social Services (17/3/3), and the IRBs of the University of Namibia (MRC/259/2017), University of California San Francisco (15-17422) and London School of Hygiene & Tropical Medicine, London, United Kingdom (10411); University of California San Francisco, San Francisco, CA (IRB No.11-05995), Makerere University, Kampala, Uganda (IRB No. 2011-0167); and Universidad Peruana Cayetano Heredia, Lima, Peru (SIDISI code 52707).

Modeling was conducted on a database of reference data from clinical specimens for which the following inclusion criteria were met: 1) available QPlex data for HRP2 and LDH antigen concentrations, 2) positive by either PCR or expert microscopy, 3) from natural clinical or asymptomatic infections in humans, and 4) ethics statements indicating IRB approval for sample collection and use. For studies with longitudinal specimen collection, only samples that were positive by microscopy or that were from a pretreatment time point were used to avoid the possibility of antigen persistence after treatment, which could bias relative concentrations of HRP2 and PfLDH. The final Pf database consisted of 1,001 samples from nine studies across 12 countries, and the final Pv database consisted of 496 samples from five studies across three countries. Deidentified data from these databases will be made available online at Harvard Dataverse: https://dataverse.harvard.edu/dataverse/PATHDXg6pd.

### Data analysis methods: estimating the relationship between antigen concentrations and the probability of RDT positivity.

For RDTs that only used a single antigen for a given species, a logistic regression model was used with probability of positivity as the independent variable and log10 antigen concentration as the dependent variable. A factor term was also included to account for the sample type (clinical, recombinant, culture, specificity). For RDTs that had two analytes for Pf (i.e., PfLDH/HRP2 tests), two separate logistic regressions models, the same as described earlier, were run, and then the outputs were combined on a surface to give an estimated probability that either (or both) test lines would be positive based on both HRP2 and PfLDH antigen concentrations. This assumes no cross-reactivity between the two test lines. A Bayesian framework was adopted for all models using the R package brms.^18^ Informative Gaussian priors with a mean of 0 and standard deviation of 3 were used for model coefficients. Each model was run for 10,000 iterations to ensure convergence. Convergence was assessed visually, using a standard convergence diagnostic called R-hat. Statistical analyses were conducted using R 4.2.1 (R Foundation for Statistical Computing, Vienna, Austria). Model predictions and 95% credible intervals were generated using the *fitted* function.

### Data analysis methods: estimating the impact of the RDTs using clinical sample data.

The values at which there was a 90% probability of positivity were extracted from the model for each RDT test line; these are referred to here as the limits of detection. Test limits of detection were then compared with antigen concentrations from the real-world samples, and the sensitivity of each test was simulated by calculating the proportion of true positives (RDT-positive and reference-positive) that have antigen concentrations greater than the limit of detection out of all samples positive by the clinical reference data. The samples from the *P. falciparum* database were overlaid on the HRP2 and PfLDH limits of detection and the samples from the *P. vivax* database were overlaid on the PvLDH limits of detection. For RDTs with two analytes for *P. falciparum*, the sensitivity was calculated as the proportion of true positive samples that had either HRP2 concentrations or PfLDH concentrations greater than their respective estimated limits of detection.

## RESULTS

### Positivity for Pf detection tests.

**Figure 1.**
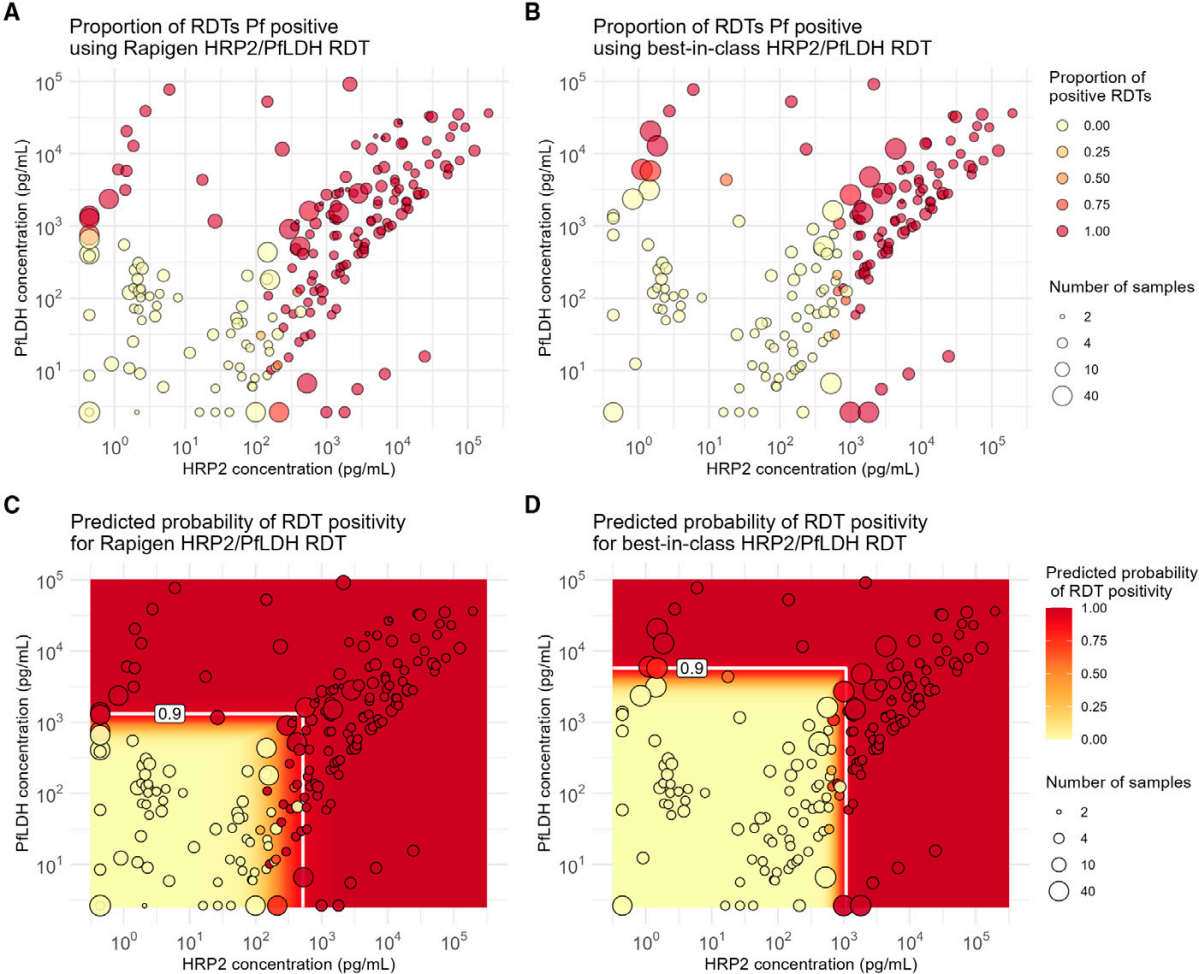
Positivity versus *Plasmodium falciparum* (Pf) antigen concentrations for Pf detection tests evaluated. (**A**) Proportion of Rapigen BIOCREDIT Malaria Ag Pf (pfLDH/HRPII) replicates testing positive and (**B**) proportion of best-in-class WHO-prequalified comparator Pf test replicates positive and plotted according to HRP2 and PfLDH concentration (picograms/milliliter). Circle size references the number of tests conducted at the concentrations of HRP2 and PfLDH in the sample, indicated by the circle placement. Heat map overlays of (**C**) Figure 1A for Rapigen BIOCREDIT Malaria Ag Pf (pLDH/HRPII) and (**D**) Figure 1B for comparator Pf test, with antigen concentrations at 90% probability of positivity for either PfLDH or HRP2, indicated by white lines. HRP2 = histidine-rich protein 2; LDH = lactate dehydrogenase.

**Figure 2.**
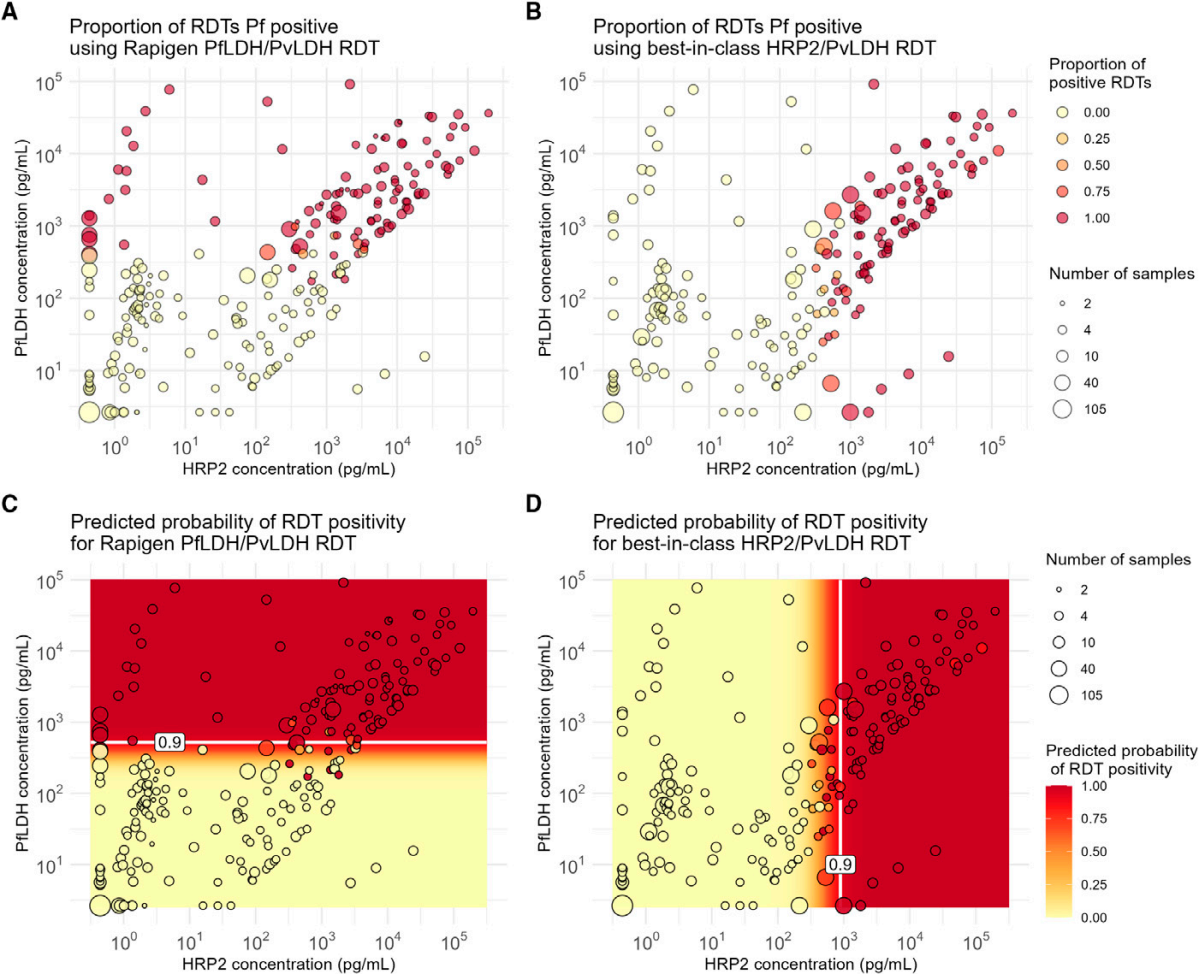
Positivity versus *Plasmodium falciparum* (Pf) antigen concentrations for Pf/*Plasmodium vivax* (Pv)-detection tests evaluated. (**A**) Proportion of Rapigen BIOCREDIT Malaria Ag Pf/Pv (pLDH/pLDH) replicates testing positive and (**B**) proportion of best-in-class WHO-prequalified comparator Pf/Pv test replicates positive, plotted according to HRP2 and PfLDH concentration (picograms/milliliter). Heat map overlays of (**C**) Figure 2A for Rapigen BIOCREDIT Malaria Ag Pf/Pv (pLDH/pLDH) and (**D**) Figure 2B for comparator Pf/Pv test, with relevant antigen concentrations at 90% probability of positivity, indicated by white lines. HRP2 = histidine-rich protein 2; LDH = lactate dehydrogenase.

**Figure 3.**
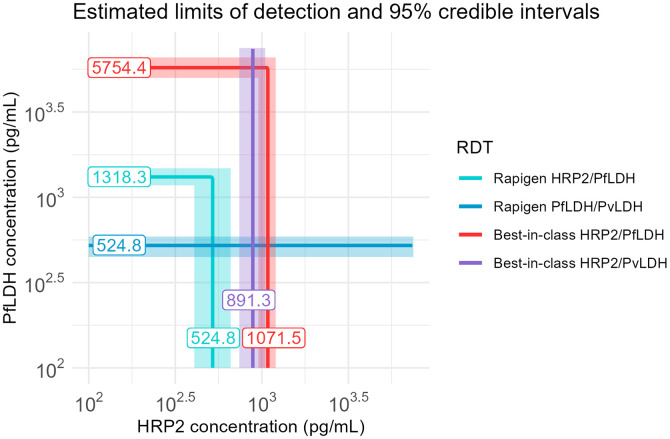
Summary of 90% probability of positivity limits. Results of modeled probability of detection are overlaid for Rapigen *Plasmodium falciparum* (Pf) (PfLDH/HRP2), Pf/Pv (PfLDH/PvLDH), and best-in-class WHO-prequalified comparator tests for Pf (HRP2/PfLDH) and Pf/Pv (HRP2/PvLDH). Antigen concentrations at the 90% probability, in pg/mL, are indicated on the line. HRP2 = histidine-rich protein 2; LDH = lactate dehydrogenase.

Replicate test results plotted according to sample concentrations of HRP2 and PfLDH proteins show a clear relationship between RDT positivity and antigen concentration across all benchmark panel members (Figure 1A and B, and Figure 2A and B). The logistic regression fits for both HRP2 and PfLDH probability of positivity versus analyte concentration indicate that the Rapigen tests can detect lower concentrations of PfLDH and HRP2 antigen. For PfLDH, the 90% probability of detection was identified at 1,318 pg/mL for the Rapigen Pf (HRPII/pLDH), 525 pg/mL for the Rapigen Pf/Pv, and 5,754 pg/mL for the best-in-class comparator test (Figures 1C and D and 2C). For HRP2, the 90% probability of detection was identified at 525 pg/mL for the Rapigen Pf (HRPII/pLDH), 1,072 pg/mL for the Pf (HRP2/PfLDH) comparator, and 891 pg/mL for the Pf/Pv (HRP2/PvLDH) comparator (Figures 1C and D and 2D). The detection of Pf by combined Pf/Pv tests showed that exclusion of either HRP2, as in the case the Rapigen Pf/Pv test, or exclusion of PfLDH, as with the comparator Pf/Pv test, limits the numbers of samples that are detected overall by HRP2 and/or PfLDH as positive for Pf (Figure 2). A graphical summary of the HRP2 and PfLDH antigen detection limits is shown in Figure 3, and limits of detection with 95% credible intervals are listed in Table 2. The largest gain in sensitivity between the comparator and the Rapigen RDTs is in detection of PfLDH.

**Table 2:** HRP2 and PfLDH antigen concentrations identified at 90% probability of positivity (and corresponding 95% credible intervals) for Rapigen and best-in-class WHO-prequalified comparator rapid diagnostic tests (RDTs)

Test	Antigen Concentration at Which Test has 90% Probability of Positivity			
	HRP2 (pg/mL)		PfLDH (pg/mL)	
	Median Estimate	95% Credible Interval	Median Estimate	95% Credible Interval
Rapigen Pf (pLDH/HRPII)	525	(407–661)	1,318	(1,175–1,479)
Rapigen Pf/Pv (pLDH/pLDH)	–	–	525	(447–589)
WHO PQ comparator HRP2/PfLDH RDT	1,072	(955–1,202)	5,754	(5,012–6,607)
WHO PQ comparator HRP2/PvLDH RDT	891	(741–1,047)	–	–

HRP2 = histadine-rich protein 2; LDH = lactate dehydrogenase; Pf = *Plasmodium falciparum*; pLDH = *Plasmodium* lactate dehydrogenase; PQ = prequalified; Pv = *Plasmodium vivax*.

### Positivity for Pv tests.

**Figure 4.**
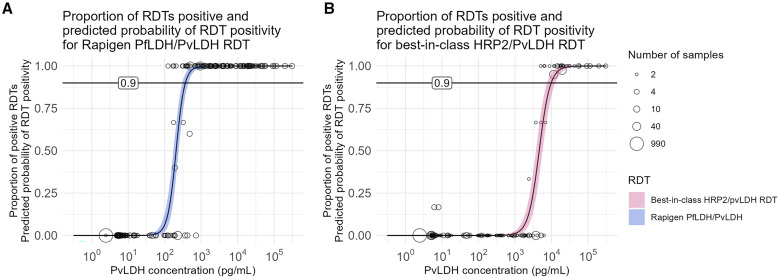
Positivity versus Pv antigen concentrations for Pf/Pv-detection tests evaluated. Proportion of A) Rapigen BIOCREDIT Malaria Ag Pf/*Plasmodium vivax* (Pv) (pLDH/pLDH) or (**B**) best-in-class comparator test replicates testing positive for PvLDH versus sample PvLDH concentration (picograms/milliliter). Circle placement indicates the proportion of samples at the given concentration testing positive, and the circle size indicates the number of samples tested at that concentration. The logistic regression fit of probability of positivity for (**A**) Rapigen (blue), and (**B**) best-in-class comparator (pink) is marked with 90% probability. HRP2 = histidine-rich protein 2; LDH = lactate dehydrogenase.

The Rapigen Pf/Pv (pLDH/pLDH) test can detect lower concentrations of PvLDH in samples compared with a best-in-class comparator Pf/Pv test. Modeled positivity versus antigen concentration indicates that the Rapigen Pf/Pv has a 90% probability of detection of 372 pg/mL, and the comparator test 90% probability of detection was found to be at 9,772 pg/mL PvLDH (Figure 4A and B). Table 3 summarizes the detection limits and corresponding credible intervals. The increase in sensitivity represents a more than 10-fold improvement in detection limit with the Rapigen Pf/Pv test compared with the best-in-class comparator.

**Table 3:** PvLDH antigen concentrations identified at 90% probability of positivity for Rapigen and best-in-class comparator rapid diagnostic test (RDT), with corresponding 95% credible intervals

Test	PvLDH Antigen Concentration (pg/mL) at Which Test Has 90% Probability of Positivity	
	Median Estimate	95% Credible Interval
Rapigen Pf/Pv (pLDH/pLDH)	372	302–447
Best-in-class HRP2/PvLDH RDT	9,772	7,586–12,883

HRP2 = histadine-rich protein 2; LDH = lactate dehydrogenase; Pf = *Plasmodium falciparum*; pLDH = *Plasmodium* lactate dehydrogenase; Pv = *Plasmodium vivax*.

### Test specificity and reactivity to *P. knowlesi* and *hrp2* or *hrp3* gene deletions.

All tests and lots used in this evaluation showed high specificity toward malaria-negative donor blood samples and high concentration of human recombinant LDH. Among the strains HB3 (*hrp2+*/*hrp3–*), D10 (*hrp2−*/*hrp3+*), and Dd2 (*hrp2–*/*hrp3+*), there were small differences in reactivity in the Rapigen tests, which detected all strains down to 200 parasites per microliter or lower, suggesting that the presence of *hrp2* or *hrp3* individual deletions still provide adequate target analytes for detection at the HRP2 line. The comparator tests preferentially detected the HRP2 over HRP3; D10 was undetectable, and Dd2 was weakly detectable at 2,000 parasites per microliter, whereas HB3 was detectable at 200 parasites per microliter or lower by the comparators. Cultured *P. knowlesi* was not detected by any test’s PfLDH or HRP2 test lines but was strongly detected by the Rapigen Pf/Pv PvLDH test line down to 125 parasites per microliter, whereas the Pf/Pv comparator was only weakly positive at 1,000 parasites per microliter.

### Performance of the RDTs on the NIBSC antigen standards.

International standards 16/376 for Pf antigen and 19/116 for Pv antigen were tested in concentrations spanning limits of detection of HRP2, PfLDH, and PvLDH of the tests evaluated. As with the other benchmarking panel members, the Rapigen tests showed marked improvements in detectable dilutions compared with the comparator WHO prequalified tests for all markers, but especially for PfLDH and PvLDH. Using the Pf standard 16/376, the Rapigen tests were able to detect at least 80% of five replicates or at least 90% of 40 replicates run at 3.9 IU/mL HRP2 and 3.9 IU/mL PfLDH (for Rapigen Pf [pLDH/HRPII]), and 1.95 IU/mL PfLDH (for Rapigen Pf/Pv [pLDH/pLDH]). Comparator tests detected at least 80% of five replicates or at least 90% of 40 replicates run at 15.6 IU/mL HRP2 (comparator HRP2/PvLDH test) and 15.6 IU/mL HRP2 and 31.3 IU/mL PfLDH (comparator HRP2/PfLDH test). Using the Pv standard 19/116, the Rapigen Pf/Pv test could detect at least 80% of five replicates at 5 IU/mL PvLDH, and the comparator could detect at least 80% of five replicates at 50 IU/mL PvLDH. Concentrations of target analytes and proportion of positive replicates at all dilutions are shown in the Supplemental Material (Tables A and B).

### Estimating malaria detection by the Rapigen and comparator tests using antigen concentration distributions of clinical samples.

**Figure 5.**
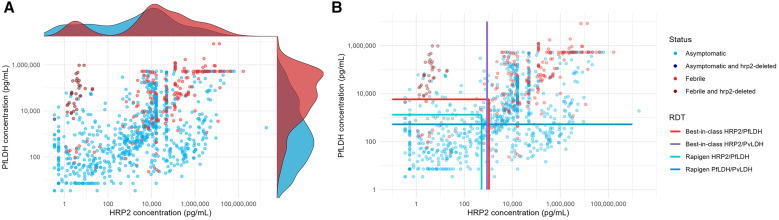
Distribution of antigen concentration in asymptomatic and febrile samples positive for *Plasmodium falciparum* (Pf) and predicted detection by Rapigen and comparator rapid diagnostic tests. (**A**) PfLDH and HRP2 concentrations are plotted for clinical samples from both symptomatic (dark red, pink) and asymptomatic (dark blue, cyan) patients positive for Pf infection by polymerase chain reaction. Points with either dark red or dark blue coloring represent samples with high probability or confirmed *hrp2* gene deletion. Above the plotted values and relative to x-axis HRP2 concentrations, curves show the HRP2 antigen distribution from febrile (red) and asymptomatic (cyan) patients. To the right of the plotted values and relative to the y-axis PfLDH concentration, curves show the PfLDH antigen distribution from febrile (red) and asymptomatic (cyan) patients. (**B**) Plotted points from panel A with overlaid lines marking 90% probability of detection concentrations for Rapigen tests (light and dark turquoise lines) and best-in-class comparator tests (red and purple lines). HRP2 = histidine-rich protein 2; LDH = lactate dehydrogenase.

The performance of the RDTs on clinical specimens was modeled by applying the 90% probability of detection concentrations identified in the benchmarking on clinical sample sets from 751 asymptomatic and 250 febrile patients with corresponding antigen concentration data. Although there is a significant overlap in distributions of HRP2 concentration between asymptomatic and febrile Pf cases, HRP2 reaches lower concentrations in asymptomatic cases. A small peak at low HRP2 concentrations (near or under clinical threshold of 6.9 pg/mL HRP2^19^) in the HRP2 concentration distribution signals the presence of 32 febrile and two asymptomatic HRP2-deleted samples (Figure 5A). The concentration of PfLDH is in general much higher among febrile cases than among asymptomatic ones, although again there is a large region of overlap. The Rapigen Pf (pLDH/HRPII) RDTs showed only a modest improvement in predicted sensitivity compared with the comparators for febrile cases but was predicted to be significantly more sensitive in detection of asymptomatic cases. Detection of Pf infection was best when both HRP2 and PfLDH were targeted. The Rapigen Pf/Pv targeting only PfLDH resulted in poorer sensitivity to the comparators that targeted HRP2, except among febrile cases carrying *hrp2*-deleted parasites (Table 4).

**Figure 6.**
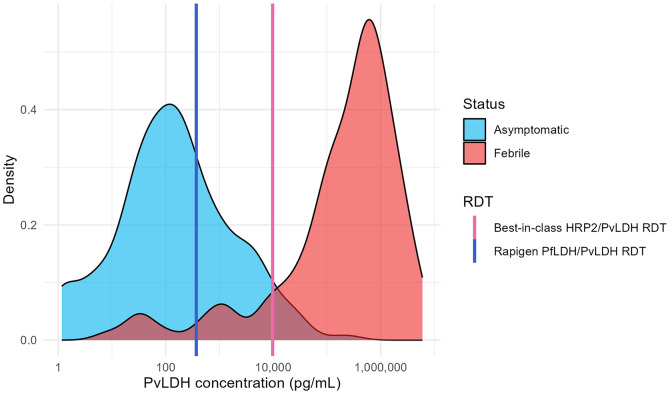
Distribution of antigen concentration in asymptomatic and febrile samples positive for *Plasmodium vivax* (Pv) and predicted detection by Rapigen and comparator rapid diagnostic tests. PvLDH antigen distributions are plotted for clinical samples from both symptomatic (red) and asymptomatic (cyan) patients positive for Pv infection by polymerase chain reaction. Lines marking PvLDH concentrations of 90% probability of detection are overlaid for Rapigen (blue) and best-in-class (pink) tests. LDH = lactate dehydrogenase.

**Table 4:** Modeled clinical performance of rapid diagnostic tests (RDTs) based on analytical detection limits and antigen concentrations of Pf-positive clinical samples

RDT	Febrile Status	Including Only *hrp2*-Present Samples		Including All Samples	
		*n*	Predicted Sensitivity and 95% CI	*n*	Predicted Sensitivity and 95% CI
Rapigen Pf (pLDH/HRPII)	Febrile	218	0.936 (0.895–0.964)	250	0.944 (0.908–0.969)
Rapigen Pf/Pv (pLDH/pLDH)	Febrile	218	0.885 (0.835–0.924)	250	0.900 (0.856–0.934)
Best-in-class HRP2/PfLDH	Febrile	218	0.927 (0.884–0.957)	250	0.932 (0.893–0.96)
Best-in-class HRP2/PvLDH	Febrile	218	0.922 (0.878–0.954)	250	0.804 (0.749–0.851)
Rapigen Pf (pLDH/HRPII)	Asymptomatic	749	0.705 (0.671–0.737)	751	0.706 (0.672–0.738)
Rapigen Pf/Pv (pLDH/pLDH)	Asymptomatic	749	0.549 (0.512–0.585)	751	0.55 (0.514–0.586)
Best-in-class HRP2/PfLDH	Asymptomatic	749	0.633 (0.597–0.667)	751	0.632 (0.597–0.667)
Best-in-class HRP2/PvLDH	Asymptomatic	749	0.611 (0.576–0.647)	751	0.610 (0.573–0.645)

HRP2 = histadine-rich protein 2; LDH = lactate dehydrogenase; Pf = *Plasmodium falciparum*; pLDH = *Plasmodium* lactate dehydrogenase; Pv = *Plasmodium vivax*.

Rapigen and best-in-class comparator RDT 90% probability of detection limits for antigens were compared with antigen concentrations in clinical samples shown in Figure 5, and predicted sensitivity was calculated. Samples were stratified by febrile and asymptomatic infection, and results were calculated both without and with (in rightmost column) *hrp2* gene deletions included.

The distribution of PvLDH in febrile and asymptomatic individuals with Pv infection showed a similar pattern of increased antigen levels among febrile cases but with less overlap between the two groups. Consequently, relative increase in sensitivity of the Rapigen Pf/Pv to the comparator was modest with febrile patients (0.955 versus 0.881), but there was a large increase in the number of asymptomatic samples with antigen levels that would be detectable, given that few were predicted to be detected by the comparator (0.326 versus 0.044) (Table 5).

**Table 5:** Modeled clinical performance of rapid diagnostic tests (RDTs) based on analytical detection limits and antigen concentrations of *Plasmodium vivax* (Pv)-positive clinical samples

RDT	Febrile Status	*n*	Predicted Sensitivity and 95% CI
Rapigen Pf/Pv (pLDH/pLDH)	Febrile	134	0.955 (0.905–0.983)
Best-in-class HRP2/PvLDH	Febrile	134	0.881 (0.813–0.930)
Rapigen Pf/Pv (pLDH/pLDH)	Asymptomatic	362	0.326 (0.278–0.377)
Best-in-class HRP2/PvLDH	Asymptomatic	362	0.044 (0.025–0.071)

HRP2 = histadine-rich protein 2; LDH = lactate dehydrogenase; Pf = *Plasmodium falciparum*; pLDH = *Plasmodium* lactate dehydrogenase.

Rapigen and best-in-class comparator RDT 90% probability of detection limits for antigens were compared against clinical samples shown in Figure 6 and the sensitivity calculated. Samples were stratified by febrile and asymptomatic infection.

## DISCUSSION

A benchmarking panel composed of frozen aliquots of recombinant antigens, NIBSC standards, cultured parasites, and clinical specimens was developed to evaluate the analytical performance of RDTs for *P. falciparum* and *P. vivax*. The use of frozen aliquots allows direct comparison of performance in a laboratory setting, with control over variables such as temperature, humidity, sample volume, and user interpretation. Although such variables are important factors in clinical settings, reducing variables for performance testing focuses the evaluation on the inherent test performance. The panel members are characterized for concentration of the target antigens relevant to the tests, and inclusion of pLDH recombinant proteins and cultured *hrp2/hrp3* deletion mutants allows evaluation of performance in the absence of HRP2 protein for a better understanding of test performance, both in situations where *hrp2* and or *hrp3* gene deletions may be increasingly prevalent or in high-transmission settings at risk of having persistent HRP2 in circulation after parasite clearance. The relationship between antigen concentration and parasite density is stronger for pLDH than for HRP2 because of its persistence after parasite clearance.^16^ The panel includes NIBSC standards, which allows for interpretation of the analytical sensitivity in terms of international units for cross-platform comparisons. Cultured strains can directly assess the reactivity toward strains with *hrp2/hrp3* mutations and toward *P. knowlesi*, which has highly variable reactivities among RDTs and may be important for speciation in co-endemic regions.^20^^–^^24^ The Rapigen PvLDH test line was found to react in a dose-dependent manner down to 125 parasites per microliter, supporting previous observations of its reactivity with *P. knowlesi*,^25^ whereas the comparator showed little reactivity. The inclusion of a broad range of antigen sources acknowledges that immunoassays may have differing reactivities toward different sources of proteins because there may be small sequence-based, conformational, or even stability differences affecting the epitopes. Although limits of detection presented were for the reactivity toward each analyte across the panel members, the limit of detection can also be stratified by source of antigen (i.e., recombinant, culture) to determine if any reactivity bias was present for specific panel components.

Included in the benchmarking panel were the First WHO International Standard for *Plasmodium falciparum* antigen (NIBSC Code: 16/376) and *Plasmodium vivax* antigen (NIBSC Code: 19/116).^26^^,^^27^ These standards offer the ability to present RDT limits of detection in standardized international units. There is an opportunity to establish target dilutions or target minimum IU/mL that manufacturers must demonstrate to regulators and procurers as part of their performance criteria. A limitation of the current standards is that they cannot discriminate the contributions of HRP2 and PfLDH concentrations to Pf positivity for RDTs with lines targeting HRP2 combined with other antigens such as PfLDH. This is particularly relevant in understanding the performance of an RDT in the context of *hrp2/hrp3* deletions.

It is possible to associate the benchmarking results to sensitivity in clinical settings through the availability of datasets with antigen concentration. On the basis of the improved detection of pLDH antigen of Pf and Pv by the Rapigen tests, a small improvement in clinical case detection is expected in which parasite densities and antigen concentrations tend to be higher. A larger relative improvement to identify subclinical cases is also expected, which will be important as regions move toward elimination or for surveillance programs. Although predicted as part of impact modeling for this study, clinical study results have indicated higher sensitivity for the Rapigen tests compared with standard tests, particularly for subclinical infections.^9^ The improved sensitivity to PfLDH is extremely important to support detection of *hrp2/hrp3* deletions. However, in RDTs that only have a PfLDH test line and do not detect HRP2, it is expected to reduce overall sensitivity against HRP2-containing Pf infections. Detection of PfLDH has performed satisfactorily against higher density panels.^28^

The selection of a broad set of samples for impact modeling may diffuse the antigen distribution patterns that may be found among different geographic regions, degrees of endemicity, and types of study, with the goal to look at overall antigenemia patterns. The distributions of the antigen concentrations showed a large overlap between febrile and asymptomatic cases but with a clear trend of higher antigen concentrations being associated with most febrile cases, except where *hrp2* deletion occurs. The predicted sensitivity among febrile versus asymptomatic cases was reflective of the trend. To explore further the predicted sensitivity with samples having higher and lower parasite density, the sensitivities were calculated using samples categorized as equal to or less than, and greater than, 200 parasites per microliter using the matched sample antigen data to define whether it would be detectable by RDT. The antigen distributions parsed by parasite density had a larger overlap than the distributions based on clinical symptoms and correspondingly had less differentiation between the relative sensitivities. However, some of the samples, although PCR positive, did not have specific parasite density information available, so this analysis is limited by proportions of the sample set that were not classified by density (Supplemental Figures A and B; Supplemental Table 2). Whether the sample sets were stratified by clinical symptoms or parasite density, the BIOCREDIT Malaria Ag Pf (pLDH/HRPII) had a higher predicted sensitivity relative to the comparator, and the performance of the BIOCREDIT Malaria Ag Pf/Pv (pLDH/pLDH) was dependent on the presence of *hrp2* deletions. When the 34 *hrp2*-deletion mutant samples were included, the pLDH-only detection was predicted to have poorer sensitivity against the whole sample set relative to a comparator that could detect HRP2 and PfLDH but had superior predicted performance relative to the comparator, which relied solely on HRP2 for Pf detection, thus missing the *hrp2*-deletion strains (Table 4).

Overall, in this laboratory analysis, the Rapigen tests performed well and had similar usability to and higher sensitivity for a given analyte than comparator RDTs, suggesting that they will have improved performance in clinical settings. This improvement in performance will be highly dependent on the distribution of antigen concentrations in the population tested. Although highly controlled laboratory settings do not robustly test usability, we found the tests to have low rates of invalid results or test defects, and they appeared to be of high quality. Although malaria RDTs are simple to use, the technique of operators may influence the overall readability and interpretation of the tests.

The two Rapigen tests have been evaluated in clinical settings.^9^^,^^29^^,^^30^ In one study, conducted in Uganda, the Rapigen BIOCREDIT Malaria Ag Pf/Pv (pLDH/pLDH), showed near equivalent sensitivity compared with a Pf/Pv test detecting HRP2 for *P. falciparum* and LDH for *P. vivax* detection.^29^ In this study *hrp*2/*hrp*3 deletions and the performance against the few Pv infections were not investigated. In a second study, conducted in Burundi, the sensitivity of the Rapigen BIOCREDIT Malaria Ag Pf(pLDH/HRPII) was higher (when either HRP2 or pLDH positivity was considered) than the HRP2-only comparator RDT against quantitative PCR, particularly for subclinical infections, despite only two *hrp3* deletion samples (from a total of 537 positive samples). However, specificity in the Rapigen test was found to be lower than the comparator. In a study conducted in Djibouti, the performance of the Rapigen BIOCREDIT Malaria Ag Pf/Pv (pLDH/pLDH) was compared retrospectively with that of two RDTs that used HRP2 for *P. falciparum* detection and LDH for *P. vivax* detection.^30^ In this study, the Rapigen test was significantly more sensitive compared with the HRP2-based tests for *P. falciparum* and also for *P. vivax* infections, as shown by both comparison in sensitivity and comparison in area-under-curve values for receiver operating characteristic analysis. Djibouti has high rates of *hrp2/hrp3* deletions and also a significant *P. vivax* infection rate.^31^^,^^32^ In countries where there is *P. vivax* and *P. falciparum* coinfection, considering the performance of the RDTs against any form of malaria may also be a useful input in selecting RDTs, especially if total malaria radical cure strategies will be considered in the future based on the observed higher risk for *P. vivax* infections in patients presenting with *P. falciparum*.^33^ Across the studies the Rapigen BIOCREDIT Malaria Ag Pf/Pv (pLDH/pLDH) RDT showed high specificity, whereas the Rapigen BIOCREDIT Malaria Ag Pf (pLDH/HRPII) RDT had significantly lower specificity compared with the other test. As described in the next section on limitations of this study, benchmarking would not identify this difference in specificity.

### Limitations of the study.

The testing and impact modeling in this study relied on data generated with an idealized workflow, from frozen specimens only, and with highly controlled sample volumes and laboratory environment, which may not represent clinical use. Variability in test response outside of the test chemistry and analyte concentration may be introduced at a clinical level and could include variable volumes from blood sample collection devices, lot differences, temperature and humidity differences, or operator vision. Such sources of variability can become integrated into clinical study performance by default or by design. Although benchmarking panels and methodology are designed to identify detection limits and focus on analytical sensitivity, other important components of test accuracy and quality are not comprehensively addressed in these studies. Notably, target analyte specificity needs to be more comprehensively evaluated with malaria-endemic region population samples and with potentially interfering substances or sample types. Cross-reactivity between analyte test lines due to high concentrations of nontarget *Plasmodium* antigen that would be associated with hyperparasitemia could be missed in this benchmarking evaluation. Given the high degree of sequence homology in LDH across all five human malaria species, inclusion of *P. malariae* and *P. ovale* antigen sources, in addition to higher concentrations of Pf and Pv antigen, will be necessary in future panels. Characterizing the reactivity of nontarget species’ antigens across different tests can identify reactivity to *P. knowlesi*, *P. malariae*, and *P. ovale* to support clinical study design, recommended follow-up testing, and treatment guidelines based on RDT results. Companion clinical studies are essential to address performance with clinical use procedures and challenges and with a breadth of samples that are not readily replicated in the laboratory setting. Clinical specificity cannot be fully interpreted using the methodology described to estimate detection based on antigen concentration alone, apart from identifying possible HRP2-persistent samples. However, these are not tested in this study because samples known to be post-treatment were excluded from the analysis described in this article. Causes for all cases of false positives cannot be revealed using antigen concentration analysis. Although inclusion of the *hrp2*-deleted samples showed some changes in the expected performance for the overall set of samples, the number of such samples was small, and samples with such deletions may not have been representative of distributions of PfLDH in the dataset.

## CONCLUSION

A method of evaluating the analytical performance of malaria RDTs is described. From this analysis, the resulting limits of detection were further applied to a dataset of clinical antigen distribution data. Both laboratory analytical sensitivity and corresponding predicted sensitivities with clinical samples are higher for the BIOCREDIT Pf and Pf/Pv RDTs from Rapigen compared with best-in-class RDT comparators. The increase in sensitivity of the Rapigen RDTs to PfLDH is likely to address to a certain extent the need for RDTs to improve sensitivity even in the presence of *hrp2* deletions. However, inclusion of PfLDH detection should still be in combination with detection of HRP2, which offers superior sensitivity relative to PfLDH for Pf.

## Supplemental Materials

10.4269/ajtmh.24-0003 Supplemental Materials
