# Supplementary material for: Analytical Sensitivity Analysis and Clinical Impact Modeling of Rapigen Rapid Diagnostic Tests for Malaria
Source: Am J Trop Med Hyg. 2024 Sep 3;111(5):956–66. doi: 10.4269/ajtmh.24-0003 (PMC11542527; doi:10.4269/ajtmh.24-0003)
Supplement: Supplemental Materials [file tpmd240003.SD1.pdf]

Supplemental Materials

Table A. Proportion positive replicate tests at different dilutions of NIBSC 16/376 International standard for Pf antigen. Proportion positive of 5 or 40 replicates shown.

Control ID	IU/mL of HRP2 and PfLDH	HRP2, pg/mL	PfLDH, pg/mL	Rapigen Pf(pLDH/HRPII)		Rapigen Pf/Pv	WHO comparator Pf(HRP2/PfLDH)		WHO comparator Pf/Pv
				HRP2 line	pfLDH line	pfLDH line	HRP2 line	PfLDH line	HRP2 line
16/376	62.5	4353	11661	1	1	1	1	1	1
16/376	31.25	1873	4760	1	1	1	0.98	0.73	1
16/376	15.63	1001	2702	1	1	1	0.9	0	1
16/376	7.81	566	1610	0.95*	1	1	0	0	0.78
16/376	3.91	295	902	1	1	1	0	0	0
16/376	1.95	146	433	0	0	0.7	0	0	0
16/376	0.98	76	205	0	0	0	0	0	0
16/376	0.49	35	94	0	0	0	0	0	0
	*2 replicates had high background and test result couldn't be determined.								

Table B. Proportion positive replicate tests at different dilutions of NIBSC 19/116 International Standard for Pv antigen.

Control	IU/mL	PvLDH, pg/mL	Proportion positive of 5 replicates	
			Rapigen Pf/Pv	WHO comparator Pf/Pv
19/116	400	99553	1	1
19/116	200	45480	1	1
19/116	100	23196	1	1
19/116	50	13506	1	1
19/116	25	4122	1	0.6
19/116	15	2534	1	0
19/116	10	1807	1	0
19/116	5	927	1	0
19/116	2.5	484	0.6	0
19/116	1	191	0.4	0
19/116	0	0	0	0
